# Behandlung von juvenilem bullösem Pemphigoid mit Lebrikizumab

**DOI:** 10.1111/ddg.15831_g

**Published:** 2025-11-14

**Authors:** Marcel Wittenberg, Farzan Solimani, Amrei Dilling, Rawan Snobar, Kamran Ghoreschi, Marisa Klemp, Kerstin Kusch

**Affiliations:** ^1^ Klinik für Dermatologie Venerologie und Allergologie Charité – Universitätsmedizin Berlin Corporate Member of Freie Universität Berlin Humboldt‐Universität zu Berlin Berlin Institute of Health, Berlin

**Keywords:** Interleukin 13, Juveniles bullöses Pemphigoid, Lebrikizumab, Monoklonale Antikörper, Pädiatrische Dermatologie, interleukin 13, juvenile bullous Pemphigoid, lebrikizumab, monoclonal antibodies, pediatric dermatology

Sehr geehrte Herausgeber,

Das juvenile bullöse Pemphigoid (BP) ist eine seltene Erkrankung mit Blasenbildung, die bei Säuglingen und Kindern im Schulalter auftritt. Wie bei der BP bei Erwachsenen lösen IgG‐Autoantikörper, die gegen BP180 und/oder BP230 gerichtet sind (zwei wichtige hemidesmosomale Komponenten), die Krankheit aus.^1^ Klinisch ähnelt die juvenile BP der BP und ist durch erythematöse Plaques, Papeln und Blasen gekennzeichnet, die von starkem Juckreiz begleitet werden, zeigt aber auch spezifische klinische Muster wie eine häufige akrale Beteiligung.^1^, ^2^ Die juvenile BP wird mit topischen und/oder systemischen Steroiden behandelt, wobei in schweren Fällen steroidsparende Wirkstoffe wie Dapson, Azathioprin oder Methotrexat erforderlich sind.^1^ Der Einsatz systemischer Immunsuppressiva ist mit dem Auftreten von Nebenwirkungen verbunden und erfordert eine ständige Überwachung der hämatologischen Parameter sowie der Leber‐ und Nierenfunktion.^3^ Monoklonale Antikörper (mAbs), die auf Zytokine abzielen, werden von den Patienten in der Regel besser vertragen und erfordern weniger häufige Blutkontrollen als orale Immunsuppressiva. Monoklonale Antikörper, die auf Typ‐2‐Zytokine abzielen, sind zur Behandlung der atopischen Dermatitis (bei Erwachsenen und Kindern) sowie der Prurigo nodularis zugelassen, jedoch nicht für die bullöse Pemphigoid‐Erkrankung, bei der Typ‐2‐Zytokine offenbar eine zentrale Rolle in der Pathogenese spielen.^4^ Der IL‐4‐Rezeptor‐alpha‐Blocker Dupilumab zeigte in Fallserien Wirksamkeit bei der BP und befindet sich in Phase III der Entwicklung.^5^ Der Anti‐IL‐13‐MAb Tralokinumab zeigte in einer kürzlich veröffentlichten Fallserie ebenfalls ermutigende Ergebnisse.^6^ Hier zeigen wir, dass Lebrikizumab, ein humanisiertes Anti‐IL‐13‐MAb, effektiv gegen juvenile BP wirkt.

Ein 16‐jähriger Junge wurde wegen des Auftretens erythematöser und juckender Plaques (VAS 9/10) am Rumpf und an den Armen sowie dem Auftreten gespannter Blasen an den Füßen in unsere Abteilung überwiesen (Abbildung 1a–d). In der Anamnese fanden sich weitere Typ‐2‐assoziierte Erkrankungen, darunter atopische Dermatitis, allergische Rhinitis und Asthma. Aufgrund des klinischen Erscheinungsbildes mit gespannten Blasen führten wir zwei Hautbiopsien für die Histologie und direkte Immunfluoreszenz (DIF) durch. Die Histologie zeigte eine milde Akanthose, eine spongiotische Dermatitis mit subepidermaler Blasenbildung und ein eosinophilen‐ und neutrophilenreiches Infiltrat. Die DIF zeigte starke lineare IgG‐Ablagerungen entlang der Basalmembran (Abbildung 1e,f). Labortests zeigten eine leichte Eosinophilie (11,5%) und stark erhöhte Gesamt‐IgE‐Werte (> 5000 kU/l), was auf eine atopische Diathese hinweist. Im Serum des Patienten konnten mittels *Enzyme‐linked Immunosorbent Assay* (ELISA) hohe Titer von Anti‐BP180‐Antikörpern (> 200 U/ml) nachgewiesen werden. Diese Ergebnisse bestätigten die Verdachtsdiagnose einer juvenilen BP bei einem Patienten mit vorbestehender atopischer Dermatitis. Es wurde zunächst eine topische Behandlung mit Betamethasonpropionat (zweimal täglich) sowie eine systemische Therapie mit Doxycyclin (100 mg täglich) in Kombination mit oralem Prednison (initial 1 mg/kg Körpergewicht) eingeleitet. Nach vierwöchiger Behandlung beobachteten wir nur eine leichte Verbesserung (VAS 8/10). Aufgrund immunologischer Überlegungen zur Immunpathogenese der BP und dem Vorhandensein begleitender Th2‐Erkrankungen sowie dem Wunsch, den Einsatz steroidsparender Mittel in jungen Jahren zu vermeiden, beschlossen wir, eine Behandlung mit Lebrikizumab (atopische Dermatitis) in Kombination mit topischem Betamethasonpropionat einzuleiten. Dieser Ansatz führte zu einem raschen Rückgang der Blasenbildung und dem Verschwinden von Hautläsionen und Juckreiz (VAS 0/10). Nach einigen Wochen fanden sich nur noch postinflammatorische, hyperpigmentierte Läsionen ohne Hinweise auf eine fortbestehende Entzündung (Abbildung 1g–i). Der Patient wurde 20 Wochen lang nachbeobachtet, ohne Anzeichen eines Rezidivs oder einer Verschlechterung. Kürzlich wurde nachgewiesen, dass eine Vorgeschichte von atopischer Dermatitis und allergischer Rhinitis das Risiko für BP erhöht, und diese Patienten benötigen häufig eine systemische Behandlung.^7^ Lebrikizumab ist zur Behandlung der atopischen Dermatitis bei Erwachsenen und Kindern ab 12 Jahren zugelassen und weist ein günstigeres Sicherheitsprofil auf als steroidsparende Wirkstoffe. Der monoklonale Antikörper bindet mit hoher Affinität und langsamer Dissoziationsrate an IL‐13, ein zentrales Zytokin in der Immunpathogenese des bullösen Pemphigoid.^4^ Interleukin‐13 ist das am stärksten exprimierte Th‐2‐assoziierte Zytokin sowohl im Blut als auch in der Haut von BP‐Patienten.^4^

**ABBILDUNG 1:**
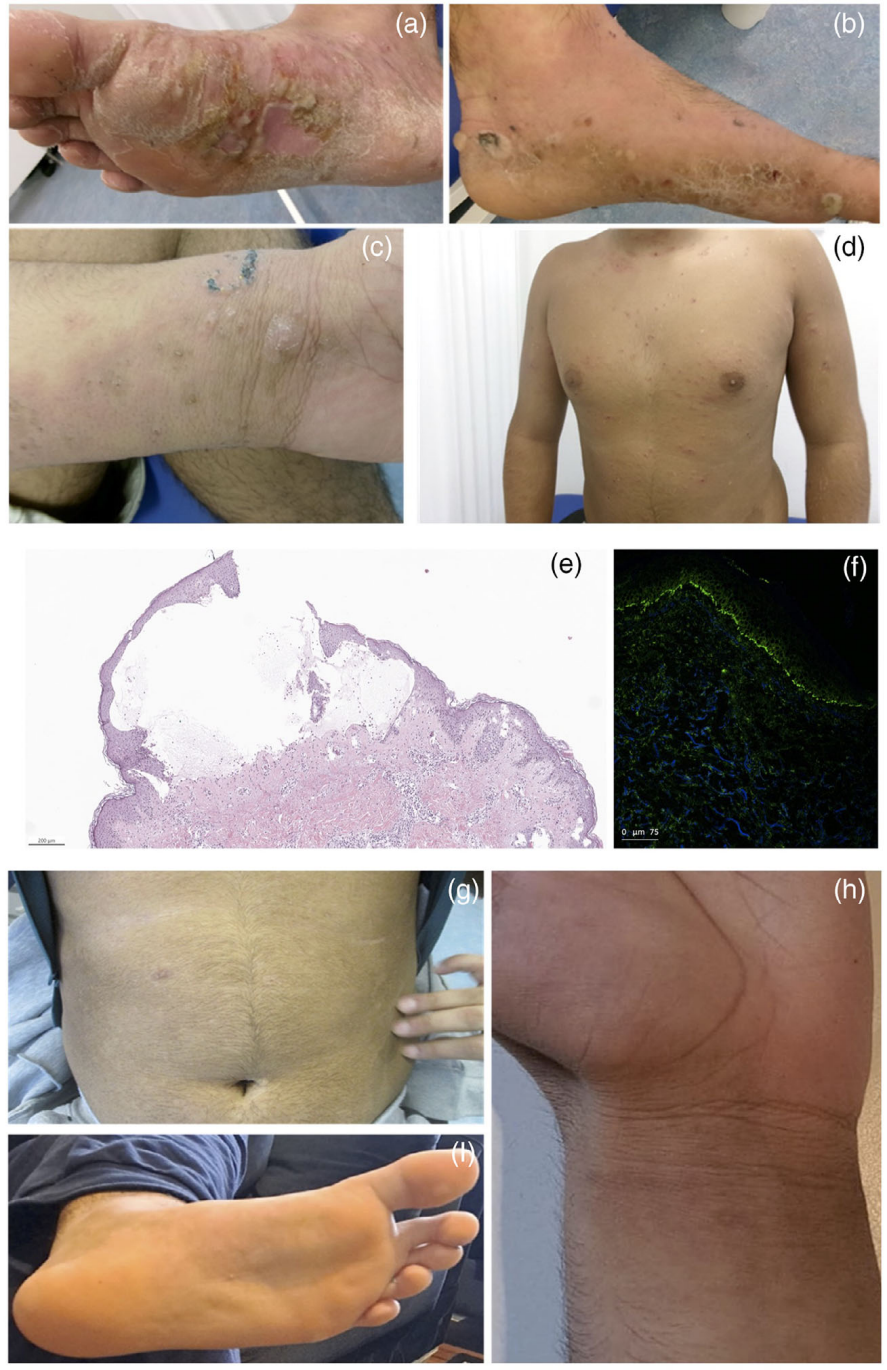
(a–d) Klinische Präsentation eines 16‐jährigen Jungen mit juvenilem bullösem Pemphigoid mit disseminierten erythematösen Papeln und Plaques sowie prallen Blasen an den Extremitäten. (e) Histologische Untersuchung zeigt das Vorhandensein einer subepidermalen Hautablösung und das Vorhandensein von Spongiose in Kombination mit einem reichen Zellinfiltrat (Hämatoxylin‐Eosin‐Färbung, Maßstabsbalken: 200 µm). (f) Direkte Immunfluoreszenz mit linearer Ablagerung von IgG entlang der Junktionszone. (g–i) Klinische Wirkung von Lebrikizumab nach 20‐wöchiger Verabreichung. Lebrikizumab führte zu einer vollständigen Remission der Hautläsionen mit minimalen postinflammatorischen Läsionen.

Dieser Bericht hebt die zentrale Bedeutung von IL‐13 in der Immunpathogenese des bullösen Pemphigoid hervor und unterstützt die Hypothese, dass IL‐13‐gerichtete Therapien zur Krankheitskontrolle beitragen könnten

## DANKSAGUNG

Open access Veröffentlichung ermöglicht und organisiert durch Projekt DEAL.

## INTERESSENKONFLIKT

Keiner.
